# Molecular identification of *Plasmodium* species responsible for malaria reveals *Plasmodium vivax* isolates in Duffy negative individuals from southwestern Nigeria

**DOI:** 10.1186/s12936-018-2588-7

**Published:** 2018-11-28

**Authors:** Mary Aigbiremo Oboh, Aida Sadikh Badiane, Godwin Ntadom, Yaye Die Ndiaye, Khadim Diongue, Mamadou Alpha Diallo, Daouda Ndiaye

**Affiliations:** 1 Parasitology and Mycology Laboratory, Université Cheikh Anta Diop, Dakar, Senegal; 2 National Malaria Elimination Programme/Epidemiology Division, Department of Public Health, Federal Ministry of Health, Abuja, Nigeria

**Keywords:** Malaria, *Plasmodium vivax*, Duffy negative, Sub-Saharan Africa, Lagos, Edo

## Abstract

**Background:**

Malaria in Nigeria is principally due to *Plasmodium falciparum* and, to a lesser extent to *Plasmodium malariae* and *Plasmodium ovale*. *Plasmodium vivax* is thought to be absent in Nigeria in particular and sub-Saharan Africa in general, due to the near fixation of the Duffy negative gene in this population. Nevertheless, there are frequent reports of *P. vivax* infection in Duffy negative individuals in the sub-region, including reports from two countries sharing border with Nigeria to the west (Republic of Benin) and east (Cameroon). Additionally, there were two cases of microscopic vivax-like malaria from Nigerian indigenous population. Hence molecular surveillance of the circulating *Plasmodium* species in two states (Lagos and Edo) of southwestern Nigeria was carried out.

**Methods:**

A cross-sectional survey between September 2016 and March 2017 was conducted. 436 febrile patients were included for the present work. Venous blood of these patients was subjected to RDT as well as microscopy. Further, parasite DNA was isolated from positive samples and PCR diagnostic was employed followed by direct sequencing of the 18S rRNA of *Plasmodium* species as well as sequencing of a portion of the promoter region of the Duffy antigen receptor for chemokines. Samples positive for *P. vivax* were re-amplified several times and finally using the High Fidelity Taq to rule out any bias introduced.

**Results:**

Of the 256 (58.7%) amplifiable malaria parasite DNA, *P. falciparum* was, as expected, the major cause of infection, either alone 85.5% (219/256; 97 from Edo and 122 from Lagos), or mixed with *P. malariae* 6.3% (16/256) or with *P. vivax* 1.6% (4/256). Only one of the five *P. vivax* isolates was found to be a single infection. DNA sequencing and subsequent alignment of the 18S rRNA of *P. vivax* with the reference strains displayed very high similarities (100%). Remarkably, the T-33C was identified in all *P. vivax* samples, thus confirming that all vivax-infected patients in the current study are Duffy negative.

**Conclusion:**

The present study gave the first molecular evidence of *P. vivax* in Nigeria in Duffy negative individuals. Though restricted to two states; Edo in South–South and Lagos in South-west Nigeria, the real burden of this species in Nigeria and sub-Saharan Africa might have been underestimated, hence there is need to put in place a country-wide, as well as a sub-Saharan Africa-wide surveillance and appropriate control measures.

**Electronic supplementary material:**

The online version of this article (10.1186/s12936-018-2588-7) contains supplementary material, which is available to authorized users.

## Background

Malaria is a principal infectious disease that continues to be a worldwide cause of mortality in endemic countries such as Nigeria. Concerted malaria intervention effort has led to a substantial gain in the reduction of global malaria cases and mortality to 41% and 62%, respectively, since 2000 [1]. However, a recent World Health Organization (WHO) report recorded that this progress has stalled and even reversed in some regions. The African Region account for about 91% of the global malaria mortality in 2016 [2]. In Nigeria, malaria remains a major public health problem and accounts for 27% of the global burden of malaria. It is estimated that 122,000 malaria associated deaths occur each year in the country [2, 3].

Malaria diagnosis remains a big challenge in malaria control and elimination and performance of different diagnostic tools differ in different epidemiological settings [4]. Microscopy which is the gold standard for malaria diagnosis can detect *Plasmodium* infections in individuals with high level of parasitaemia, however parasite detection in individuals carrying low parasite density can be challenging [5], thus emphasizing the need for more sensitive diagnostic methods. Needless to mention misinterpretation of blood smears by microscopists, inability to detect mixed species infection (as well as distinguish *P. ovale* from *P. vivax*) [6, 7], lower detection limit of parasites (between 4 and 20 parasites/µl for an expert microscopist and 50–100 parasites/µl for an average microscopist) [8, 9], and unstable power supply [10], are all drawbacks of microscopy.

In recent times, various molecular techniques have been used to detect *Plasmodium* species, with polymerase chain reaction (PCR) being the most frequently used method [11–13]. Additionally, it has also been helpful in revealing the high prevalence of mixed infections [14–17] as well as the detection of parasites otherwise not detected in the peripheral blood circulation due to sequestration.

Of the five *Plasmodium* species, infecting humans [18], *P. falciparum* is the most widely spread in Africa and causes the most severe form of the disease worldwide [2]. Hence, most malaria interventions in Africa have focused on *P. falciparum*. This unalloyed attention to falciparum malaria control interventions should be looked into as a result of the following reasons: (i) vivax malaria (as well as *P. ovale*) is less flexible to manage and may survive for a prolonged period as a result of the hypnozoite stage, thus serving as a reservoir of infection [19], (ii) *P. vivax* has a larger global distribution, and the number of people at risk of contracting it is more than that of *P. falciparum.* Hence, its incursion into sub-Saharan Africa (SSA) gives it a much wider distribution from a global perspective [20], (iii) lastly it can also provoke severe symptoms in infected patients [21].

*Plasmodium vivax* malaria has been presumed absent in SSA due to the fact that the Duffy antigen, the erythrocyte receptor for *P. vivax* merozoite invasion, is not expressed on the red blood cells of most populations in SSA, as revealed by the seminal work of Miller and colleagues, therefore are thought to be protected against vivax malaria [22]. Although a very recent findings point to another receptor on the recticulocyte-the transferrin receptor 1 as a specific *P. vivax* receptor [23]. The protein encoded by the DARC-coding gene (or ‘Duffy antigen receptor for chemokines’, DARC) is a glycosylated membrane protein that is located on the long arm of chromosome 1 (1.q22–1.q23). The expression of the DARC-coding gene on erythrocytes is connected to a single nucleotide polymorphism (SNP) (− 33T>C) in the promoter region of the DARC-coding gene on chromosome 1. In homozygous carriers (− 33CC) the DARC-coding gene on the erythrocytes is not expressed and as such are referred to as Duffy-negative [24, 25]. *Plasmodium vivax* is predominantly found in Asia, Latin America and the Horn of Africa where the majority of the population is Duffy positive [26]. Recent studies indicate that vivax malaria may be found in SSA, in Duffy positive, but also in Duffy negative individuals, or in individuals whose DARC status was not characterized [24, 26–37]. Although, recent evidence abounds [38, 39] supporting the origin of *P. vivax* from vivax-like malaria from Africa in non-human primates. However, some scientists have opined that the source of vivax malaria in SSA could be from the different migrational pattern especially along trade pathway [40]. Although, all of the aforementioned hypotheses seem credible, the precise route of entry or exit of *P. vivax* to/from Africa need further detailed study. Nevertheless, it seems more than one of the presumptions might be at play here.

Two things are at play from the above: *vivax*-infected Duffy positive individuals serving as reservoir of infection for the Duffy negative individuals [24]; and the possibility of the development of alternative receptors (such as the newly discovered transferrin 1 receptor) other than the DARC-coding protein on the surface of the erythrocytes for binding and invasion of RBCs by *P. vivax* [23, 36].

Studies conducted in the northern part of Nigeria attempting to characterize Duffy blood groups estimated 19.2% and 5.6% of the general population and pregnant women, respectively, to be Duffy positive [25, 41], thus providing a reservoir for vivax malaria, which could then be transmitted to Duffy negative people. Of special importance is the detection of vivax malaria in the Republic of Benin and Cameroon, both of which share border with Nigeria to the west and east, respectively [29, 32, 33].

In Nigeria, *P. falciparum* accounts for > 95% of malaria infection, while *P. malariae* and *P. ovale* are estimated to be responsible for < 5% of infection [1, 2]. To date, available data in Nigeria associated with *P. vivax* points to the case of a pregnant Nigerian female, visiting the country briefly from Italy where she resides and was diagnosed of mixed vivax infection [42], and other microscopically positive cases unconfirmed by any molecular technique [43, 44]. Additionally, in order to make informed epidemiological decisions, an accurate documentation of mixed species is important. Furthermore, with the observed reduction in the burden of falciparum malaria, there should be a shift of focus or effort concerted in controlling non-falciparum malaria, accounting for about four hundred million cases world-wide [21].

This study was designed to confirm the different species detected by RDT and microscopy using PCR as the gold standard, to sequence the *P. vivax* isolates in the study areas, and to determine the DARC status of *P. vivax*-infected patients.

## Methods

### Study sites

Samples were collected from Lagos and Edo states, both of which are located on the western and southern part of Nigeria respectively. Lagos state shares a border with the Republic of Benin. In Lagos state, samples were collected from four different Local Government Areas (LGAs) namely Eti-osa (06°26′N 003°29′E), Ibeju (06°26′N 003°56′E), Kosofe (06°28′N 003°22′E) and Ikorodu (06°33′N 003°35′E) LGA, while in Edo state, samples were collected from two LGAs: Oredo (06°19I′N 5°34′E) and Ikpoba Okha (06°16′N 5°68′E).

Lagos state is hypo-endemic in most part with a 1.9% prevalence rate in children age 6–59 months [2, 45]. This in part is due to the expansion of insecticide-treated nets (ITNs) coverage, occurrence of indoor residual spraying (IRS) in many of its LGAs [43, 46].

Edo state on the other hand is meso-endemic with a prevalence rate of 35% in children age 6–59 months [2, 45, 47]. This meso-endemic nature on the other hand has been shown to be due to low utilization of malaria control interventions such as ITNs and intermittent preventive therapy (IPT) among the population [48].

Generally, the rainfall pattern of the two states has little or no variation with an annual rainfall of 1400–1800 mm and a short break called “August break’. There are two climatic conditions predominant in both study locations, the dry season (lasting from November to March) and the wet season (from April to October), with a temperature range of 30–38 °C [49]. Malaria transmission normally occurs throughout the year with its peak transmission occurring during the raining season [46, 47] and mostly transmitted by *Anopheles gambiae* sensu stricto (s.s.) and *Anopheles funestus* s.s [2, 50, 51].

### Study design

The study was a cross-sectional investigation involving patients presenting clinical symptoms of malaria and visiting any of the various hospitals in both study sites between September 2016 to March 2017. The inclusion criteria were patients that were ≥ 2 years of age, clinical symptoms of malaria detected by a febrile condition of ≥ 37.5 °C while the non-inclusion criteria were being pregnant and having complicated infections.

**Fig. 1:**
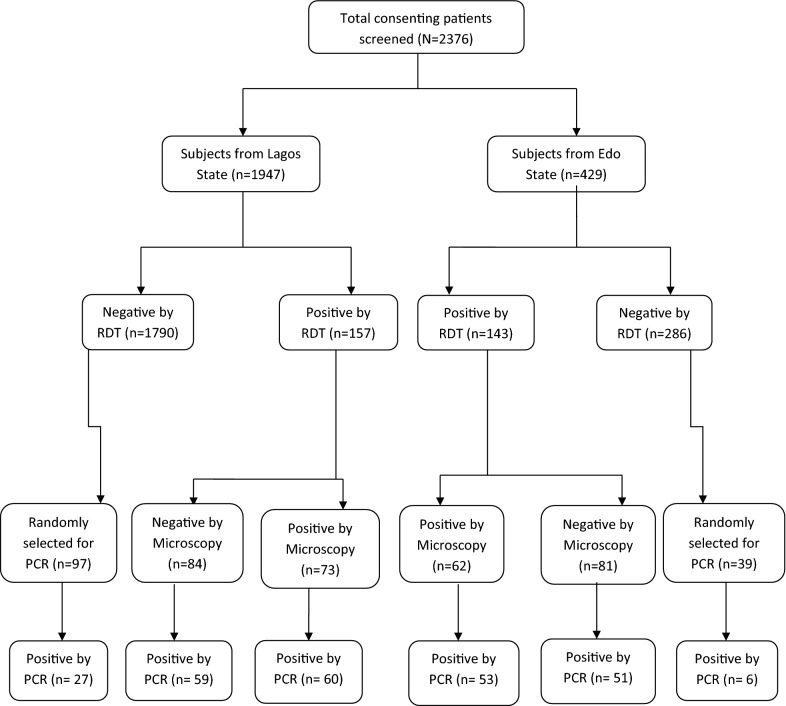
Flow chart of sample processing. A total of 300 samples were positive by RDT out of which only 135 and 256 turned out positive by microscopy and PCR respectively. From the pool of 136 randomly selected negative samples, 33 more turned out positive by PCR

Employing convenience sampling, a total of 2376 consenting patients were recruited from all study sites after detailed briefing of the purpose of the study. All study subjects were initially screened using *P. falciparum* specific HRP2 RDT kits (Care Start^®^, Access Bio Inc, Batch number M014L04–M014M10 henceforth referred to as just RDT). Those found positive by this initial diagnostic test were further screened by microscopy and subsequently by nested PCR. From the pool of samples that were negative by both RDT and microscopy, 136 were randomly selected by taking every fifth, seventh or tenth negative samples (depending on the study location and sample size) and were all subjected to PCR (Fig. 1). During this study, care providers of each hospital assumed absolute responsibility for patient care; all decisions relating to diagnosis and treatment were done irrespective of research protocol and outcome.

### Sample collection and preparation

Venous blood collected from each consenting/assenting patients was kept in ethylene diamine tetra acetic acid (EDTA) containers corresponding to a patient’s unique identity for RDT and subsequent microscopy and PCR analysis. Following the manufacturer’s instruction, Care Start^®^ *P.f* (Access Bio Inc, USA), which only detects the histidine-rich protein 2 (HRP-2) of *P. falciparum,* was used to carry out a rapid diagnosis following the manufacturer’s instruction, and those samples found positive by this preliminary test were processed further.

### Parasitological examination and parasite density determination

Preparation of thin and thick blood films and determination of parasites density followed a previous protocol [52]. Briefly, thin and thick smears were made for each sample collected in EDTA, the thin film was fixed in methanol and both films were dried and stained with Giemsa and examined under oil immersion at the Nigerian Institute of Medical Research. Each positive thick smear was counted against a minimum of 500 leucocytes and evaluation of parasite density done using the estimated 8000 white blood cells (WBCs)/μl of whole blood while the thin smear was used for species identification, following the formula below.

$${\text{PD}}\, = \,\left( {{\text{NPC}} \times 8000} \right)/{\text{N}}_{\text{WBCs}}$$

PD = parasite density; NPC = number of parasites counted; N_WBCs_ = number of white blood cells (fixed to 500).

### DNA extraction

Blood spots from positive (RDT and/or microscopy) as well as negative (few selected RDT and microscopy) samples collected from consenting patients were made on Whatmann^®^ filter paper (GE Healthcare, Life Sciences) and allowed to dry at room temperature. Each filter paper was kept in a zip-locked sachet with silica gel, stored at room temperature (or at − 20 °C where available) and transferred to Parasitology and Mycology laboratory, Aristide Le Dantec University Hospital, Dakar, Senegal. Following the manufacturer’s instruction, DNA was extracted from three 3 mm punches of DBS using the QIAmp DNA Blood Mini Kit (Qiagen^®^, Hilden Germany) and eluted in a 100 µl final volume and stored at − 20 °C until ready for use.

### PCR amplification of the 18S rRNA of *Plasmodium* species and the Duffy antigen receptor for chemokines of human

**Fig. 2:**
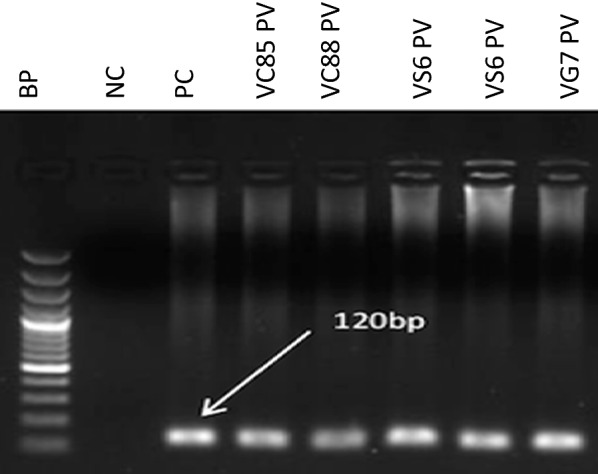
Gel documentation of *P. vivax* species detected in the study area: First well: DNA base pair ladder, well 2: PC-*P. vivax* positive control, well 3–7: *P. vivax* samples

In order to compare the other two diagnostic tool with PCR as well as molecularly identify the circulating species in the study area, a primary and nested PCR was carried out to differentiate all *Plasmodium* spp. Both PCR targeted the 18 subunit ribonucleic acid (18S rRNA) for both the genus as well as species using previously described protocol [53]. PCR amplification was carried out with 1 µl extracted DNA and 2 µl of next 1 amplicon for the primary and nested PCR respectively using the Gotaq Green Mater mix (Promega) with detailed constituents outlined in Additional file 1: Table S1. Each sample detected as *P. vivax* was extracted and re-amplified 3 times in order to reconfirm that they are *P. vivax* following same protocol as above. Additionally, all *P. vivax* isolates were re-extracted and -amplified using the High Fidelity fusion Taq (New England Biolab, Inqaba biotec, Pretoria South Africa) to negate any bias likely to be introduced during the amplification process. Clinical isolates of *P. falciparum*, *P. malariae, P. ovale* and *P. vivax* (PCR amplified and confirmed by sequencing) and sterile distilled water were used as the positive and negative controls respectively to validate the nested PCR approach. PCR products in the DNA of a patient’s specimen corresponding to 205 bp (*P. falciparum*), 144 bp (*P. malariae*), 800 bp (*P. ovale*) and 120 bp (*P. vivax*) were confirmed as positive for the respective species (Fig. 2).

With a view to ascertaining the DARC status of the *P. vivax* infected individuals, isolated DNA from *P. vivax* infected samples were used, since it also contains human DNA. Using already established primers and protocols [54], a 630 bp PCR fragment covering the promoter region was amplified. This technique has been utilized since it is established that sequencing of this promoter region of the human Duffy gene with regards to the − 33rd nucleotide position can proficiently determine the Duffy status [32, 33, 35, 36, 55]. The appearance of the C-nucleotide in a single peak in the chromatogram at the − 33rd position downstream the promoter region depicts entirely the lack of expression of the Duffy gene on the erythrocytes which is also denoted as the FY*O homozygote genotype, while the detection of both the C and T nucleotide at same position stipulates the heterozygote condition and a single peak with the T-nucleotide indicate the absence of the FY*O genotype [33, 54]. Primer details and PCR conditions are located in Additional file 2: Table S2.

### Sequencing of PCR products and multiple sequence alignment

In order to validate the different species identified by PCR in gel electrophoresis, the 18S rRNA gene of *P*. *vivax* and the DARC-coding gene of the *P. vivax* infected individuals were sequenced commercially by Inqaba Biotec, West Africa Ltd, Pretoria (Quote No. NG2018/13544). Request was also made for purification of PCR products prior sequencing. For each fragment, sequencing was carried out from both the 5′ and 3′ directions (2× coverage). Consensus sequences from both the forward and reverse sequences were generated using the BioEdit computer software. Multiple sequence alignment of the 18S rRNA of the vivax isolates and the human Duffy gene was done with BioEdit. Sequences generated from the current study were aligned with their respective reference sequences; *P. vivax* (SAL-1 accession number U03079.1) and accession number NG_011626.30 for the Duffy gene. All references were retrieved from the NCBI website (https://www.ncbi.nlm.nih.gov) by employing the BLAST search.

### Statistical analysis

All other data aside the sequences were analysed using Statistical Package for Social Sciences (SPSS) version 21.0 (SPSS, Inc. Chicago IL, USA). Furthermore, the performance of each diagnostic test method was calculated by means of sensitivity, specificity, positive predictive value (PPV), and negative predictive value (NPV) using nested PCR as a gold standard. Kappa’s statistics was used to test the level of agreement between the diagnostic tools. *P *< 0.05 was considered statistically significant.

For ease of cartographic representation of the diversity of mixed species originating from the different study areas, Eti-Osa and Ibeju were grouped as Lagos Island-owing to their location on the part of Lagos state surrounded by the Lagos lagoon and extending into the Atlantic ocean while Oredo and Ikpoba-Okha were grouped as Edo due to their supposed boundary sharing on the western part of Ikpoba-Okha.

## Results

From the overall 2376 surveyed study subjects, 436 were included for this study with 39.2% (171/436) of them being from Oredo LGA. The mean age of the study population was 23 years (with a range of 1–85 years). The sex ratio of the overall study population was 0.57 (Table 1).

**Table 1:** Characteristics of the study population in the six different study sites

	Eti-Osa	Ibeju	Kosofe	Ikorodu	Oredo	Ikpoba-Okha	Total
Number	44	57	71	82	171	11	436
Percentage (%)	10.09	13.07	16.28	18.81	39.22	2.53	100
Age							
Mean	22	18	25	17	26	33	
Range	[4–62]	[2–65]	[2–85]	[1–67]	[1–85]	[10–79]	
Sex							
Male	28	19	36	38	73	3	197
Female	16	38	35	44	98	8	239

Number: the figure of subject surveyed in each study sub-location

As described above (Fig. 1), 436 selected samples (300 samples positive by RDT; 135 by microscopy; 165 negative by microscopy, and 136 randomly selected among those negative by RDT) were tested with molecular methods. Of the 300 infections detected as positive by RDT, only 135 were positive by microscopy while the remaining (165) were considered false positives. Similarly, of this same 300 RDT-positive samples further subjected to PCR, only 223 turned out to be positive, while the other 77 were considered false positives by RDT.

Similarly, of the 135 samples detected as positive by microscopy, only 113 were truly positive (60 from Lagos and 53 from Edo) as detected by PCR while the remaining 22 were false positive. Hence, the sensitivity of microscopy was higher (83.7%) than its specificity (52.5%) (Fig. 1; Table 2).

**Table 2:** Evaluation of the diagnostic performance of microscopy and RDT versus PCR

	PCR		Sensitivity (%)	Specificity (%)	PPV (%)	NPV (%)	Kappa’s test	*P* value
	Positive	Negative						
Microscopy								
Positive	113	22	83.7	52.5	44.1	87.8	0.09	0.00
Negative	143	158						
RDT								
Positive	223	77	74.3	74.5	87.1	57.2	0.18	0.00
Negative	33	103						

*PPV* positive predictive value, *NPV* negative predictive value

However, a reverse pattern was observed in the probability of a positive or negative isolate being correctly identified as such. With RDT, the chances of a positive samples turning out positive by PCR, also known as the positive predictive value (PPV) was very high (87.1%) while the chances of it being picked as truly negative; negative predictive value (NPV) was very low (57.2%). However, the reverse was the case for microscopy, with the NPV being higher (87.8%) than the PPV (44.1%). Assessing the level of agreement using PCR as the gold standard showed that RDT has a weak agreement (Kappa’s statistic = 0.18) with PCR while microscopy shows no agreement (Table 2).

Of the 436 samples collected, the *Plasmodium* 18S rRNA was amplifiable in 58.7% (256/436), 110 from Edo and 146 from Lagos. Majority of the infections were due to *P. falciparum* either as single infection 85.5% (219/256; 97 from Edo and 122 from Lagos), or mixed with *P. malariae* 6.3% (16/256), *P. vivax,* 1.6% (4/256) or with *P. ovale* 1.2% (3/256). *Plasmodium malariae* single infection accounted for about 4.7% (12/256) of the total positive samples. Only one of the five *P. vivax* isolates was found to be a single infection and only one isolates (0.4%) was found with triple infections of *P. falciparum, P. malariae* and *P. vivax*. It is interesting to note that all of the *P. vivax* sample were from microscopically negative slide detected both in Nigeria and Senegal by a trained and WHO level one microscopist respectively.

**Fig. 3:**
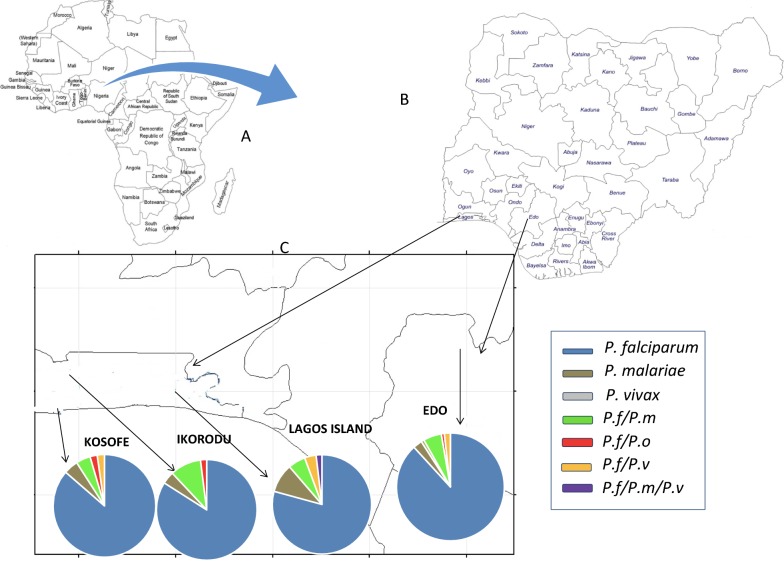
Map of **a** Africa showing the location of Nigeria with Benin and Cameroon bordering Nigeria to the west and east respectively. **b** Nigeria pointing to the two states (Lagos and Edo) where study was conducted and **c** study sites showing the diversity of the *Plasmodium* species present in each site (grouped site)

With regards to the diversity of *Plasmodium* species, Edo state has the highest diversity of mixed infection with all species mono infection (except *P. ovale*) conspicuously represented in addition to mixed *P. falciparum*/*P. malariae*, *P. falciparum/P. ovale* and *P. falciparum*/*P. vivax* infection. This is followed by Kosofe and Lagos Island with *P. falciparum*, *P. malariae* mono infections as well *as P. falciparum*/*P. malariae* mixed infections. The least diversed site by species composition is seen in Ikorodu (Fig. 3).

**Fig. 4:**
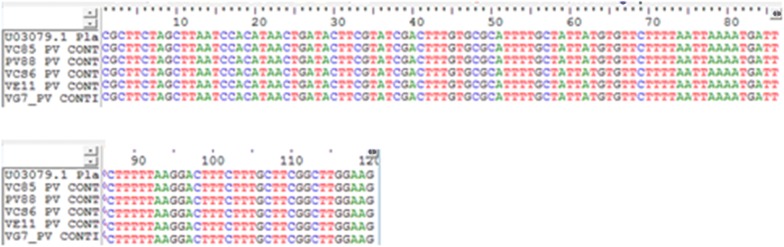
Multiple sequence alignment of 18S rRNA of Nigerian. *Plasmodium vivax* isolates with the 18S rRNA of *P. vivax* SAL-1 strain

In order to authenticate the PCR results with regards to vivax infections, sequencing of the 18S rRNA was carried out on all *P. vivax* isolates (4 mixed and 1 mono infection). The sequences of the 18S rRNA genes, accompanied by multiple sequence alignment with its reference sequences (U03079.1) showed perfect homology (100% similarity) (Fig. 4). The newly generated sequences of the 18S rRNA genes of all four species will be deposited in the GenBank (MK131265–MK131269).

**Fig. 5:**
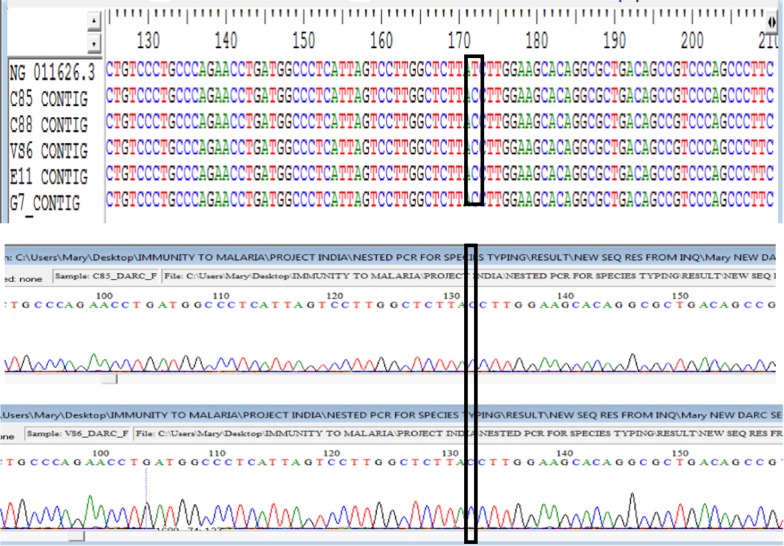
Sequence alignment of human Duffy gene of *P. vivax* patients and its accompanying chromatogram with the reference positive strain (NG_011626.3). The T-33C mutation which depicts Duffy negative is shown in the rectangle enclosing the cytosine

The sequences of the Duffy gene were trimmed to 620 bp which is more than sufficient to cover the − 33rd nucleotide position. The − 33C mutation was found in all *vivax* infecting patients. Diligently, each chromatogram was manually visualized in order to determine if the Duffy genes are homozygote at the − 33rd nucleotide position downstream the promoter region (which will be displayed as a single C peak) or heterozygote with the occurrence of double T and C peak. A single peak of C-nucleotide was found at the − 33rd position of the Duffy gene promoter region in both the forward and reverse direction of the sequences (2× coverage) (Fig. 5), indicating that all *P. vivax* infected individuals are homozygous Duffy negative, hence there is no expression of the gene on the erythrocytes of the infected individuals. The newly generated sequences of the Duffy promoter region have been submitted to Genbank (MK135816–MK135820).

## Discussion

This study assessed the performance of three diagnostic tools in detecting malaria from six endemic settings with varying levels of transmission in Nigeria. Analysis from this study provides the rate of false positives, false negatives as well as the confirmation of *P. vivax* in Duffy negative individuals, all of which have implications in malaria control programmes.

Firstly, with regards to the false negative rate, an appreciable number of the sub-microscopy carriers (33/136 randomly selected samples detected as negative both by RDT and microscopy) were not treated as they were wrongly diagnosed as negative both by RDT and microscopy. The failure of RDT to detect these individuals as positive on the one hand, could be due to low levels of target *Pf*HRP2 antigen or even a deletion of it entirely from the isolate [56–58] identified as negative. Failure by microscopy on the other hand, could be due to low level of parasitaemia. All of this group of untreated individuals pose a huge challenge to malaria control effort as they subsequently serve as reservoir of infection for continuous transmission [58–60]. Even with mass screening, when sensitive technique is not being employed, this group will still go undetected, thus necessitating the use of highly sensitive and specific techniques such as PCR that is capable of detecting very low levels of parasites missed both by RDT and microscopy as has been shown by various studies [4, 18, 32, 58, 61]. Contrary to this study, the sub-microscopic prevalence of *P. falciparum* of other studies was quite lower than what was obtained here [18, 62], this however, could be due to the experience of the microscopist who read the slides (as the slides were read by a WHO level 1 microscopist), difference in the transmission intensity as well as intervention implementation, which could both (transmission and intervention) influence the burden of parasite carriage in various epidemiological settings.

In the current study, nested PCR targeting the 18S rRNA gene of *Plasmodium* species was used to detect single and mixed infections. The study reveals the dominance of *P. falciparum* both as single or mixed infection with *P. malariae*, *P. vivax* or *P. ovale*. This elevated prevalence of *P. falciparum* among the overall study population as well as in previous studies from Nigeria [1, 2, 43, 63] seems to substantiate the focused attention of malaria control strategies towards this species. Of all the sites surveyed, Oredo and Ikpoba-Okha regrouped as Edo state displayed a high diversity of mixed malaria species with the exception of *P. ovale* mono infection. Cases of mixed *P. falciparum* and *P. vivax* infection have been found to lead to significant severe malaria in Australia as well as in Asia [64, 65]. Additionally, the occurrence of sixteen and three *P. malariae* and *P. ovale* mixed (mainly with *P. falciparum*) infections respectively might have been underestimated by microscopy in this current study. This is because *P. ovale* infect reticulocytes which happens to be also among the stage parasitized by *P. falciparum*. This could have resulted in the mis-identification observed in the current findings [66]. The co-infection of *P. ovale* and *P. falciparum* in this present study is in agreement with findings from other parts of Nigeria [30, 43] where both species have been found to co-infect individuals. The occurrence of such mixed species should be taken into consideration when planning for malaria control interventions.

Interestingly, the present study report the first molecular evidence of *P. vivax* from individuals residing in both Lagos and Edo state of Nigeria. Corroborating the PCR outcomes is the result obtained from the sequencing of *P. vivax* amplicons and subsequent alignment with the SAL-1 reference strain which gave a perfect homology (100% similarity) with its reference strain, suggesting high reliability of molecular techniques as confirmatory diagnostic tool. Before now, the reports of *P. vivax* connected to Nigeria are those of a traveller [42] as well as microscopically detected *P. vivax*-like malaria [43, 44]. Wether the *P. vivax* detected in the traveller who visited Nigeria originated from Nigeria or it was a relapse of *P. vivax*, since it is known to cause relapse as a result of the dormant hypnozoite stage weeks or even months after treatment [67] is unknown. Although, this is, in our knowledge, the first molecular confirmed incidence of *P. vivax* in Nigeria, it is however, not surprising as it has been detected elsewhere in SSA where it was thought to be initially absent [24, 28, 35, 37] due to the high prevalence and near fixation of the Duffy-negative gene in Africa which is thought to confer resistant against *P. vivax* infection [22, 55].

Additionally, the two dominant vector species responsible for malaria transmission in Nigeria-*An. gambiae* and *An. funestus* [2] have been implicated in their ability to transmit *P. vivax* malaria in a study carried out in East Africa [37], it therefore stands to reason that, it is likely that *P. vivax* can be successfully transmitted from neighbouring African countries especially given the fact that cases of *P. vivax* have been reported from the Republic of Benin [29] and Cameroon two countries sharing boundaries with Nigeria to the west and east, respectively. Hence, the defensive barrier hypothesis against *P. vivax* infection due to the near fixation of the Duffy-negative gene in the African population [22] should be looked into.

Interestingly, multiple sequence alignment of the DARC-coding gene of the nine *P. vivax* infected patients revealed all of them to be homozygous Duffy negative with a single C-nucleotide peak. The present findings taken together with outcomes from similar studies in other Africa countries suggest that *P. vivax* might be evolving and adapting new strategies in infecting Duffy-negative individuals, just as similar adaptive strategy was observed in the simian malaria parasite-*Plasmodium knowlesi* now infecting humans [18].

An important limitation of this study is the inability to link *P. vivax* mono and mixed infection with disease clinical outcomes as studies from Asia in both *P. vivax* mono and mixed infection (*P. vivax*/*P. falciparum*) [64, 65, 68] revealed severe clinical outcomes in both cases. Another limitation is the fact that this survey is restricted to the south-western region, (two out of the 36 states in Nigeria), hence does not give a full representation of the burden of *P. vivax* in Nigeria. Additionally, though the study was conducted in core areas inhabited by indigenes, the exact travel history of those with *P. vivax* could not be ascertained, whether they had previously visited a country with circulation of *vivax* malaria is uncertain. Therefore, there is need for a country-wide survey to ascertain the burden of *P. vivax* as well as mixed malaria infection in order to put in place adequate control measures.

## Conclusion

Given the fact that Nigeria accounts for 27% of the global malaria burden mostly due to *P. falciparum* [2], the addition of *P. vivax* to this, will ultimately be a daunting task in the country’s struggle to control malaria. Therefore, the National Malaria Control Programme should initiate a country-wide epidemiological survey of all the circulating species of *Plasmodium* using very sensitive diagnostic techniques such as PCR and plan control intervention accordingly.

## Additional files

**Additional file 1: Table S1.** PCR protocol and cycling conditions used for malaria diagnosis *of P. falciparum, P. malariae*, *P. ovale* and *P. vivax.*

**Additional file 2: Table S2.** Primer sequence, PCR protocol and cycling conditions used for Human Duffy antigen receptor for chemokines.

## Abbreviations

DNA: deoxyribonucleic acid
PCR: polymerase chain reaction
rRNA: ribosomal ribonucleic acid
RDT: rapid diagnostic test
PvMSP1: *Plasmodium vivax* merozoites surface protein 1
WHO: World Health Organization
DARC: Duffy antigen receptor for chemokines
RBC: red blood cell
WBC: white blood cell
LGA: local government area
PPV: positive predictive value
NPV: negative predictive value
IPT: intermittent preventive therapy
EDTA: ethylene diamine tetra acetic acid
HRP II: histidine rich protein II
DBS: dried blood spot
SPSS: statistical software for social sciences
